# Integrated Cytokine and Immune Cell Profiling Reveals a Distinct Immune Signature Associated with High-Altitude Pulmonary Edema

**DOI:** 10.3390/ijms27177787

**Published:** 2026-08-31

**Authors:** Kanika Singh, Manzoor Ali, Krishna Kumar G, Tsering Palmo, Swati Kumari, Tashi Thinlas, Qadar Pasha, Brian B. Graham, Aastha Mishra, Rahul Kumar

**Affiliations:** 1 Genomics and Genome Biology Unit, CSIR-Institute of Genomics and Integrative Biology, Mathura Road, Delhi 110025, India; kanika0298@gmail.com (K.S.); raufali172@gmail.com (M.A.); krishnakumargm2208@gmail.com (K.K.G.); palmo1851@gmail.com (T.P.); kswati503@gmail.com (S.K.); 2 Academy of Scientific and Innovative Research (AcSIR), Ghaziabad 201002, India; 3 Department of Medicine, Sonam Norboo Memorial Hospital, Leh 194101, India; tashithinlas@gmail.com; 4 Institute of Hypoxia Research, Delhi 110067, India; pashamaq53@gmail.com; 5 Lung Biology Center, Zuckerberg San Francisco General Hospital, San Francisco, CA 94110, USA; brian.graham@ucsf.edu; 6 Department of Medicine, University of California San Francisco, San Francisco, CA 94143, USA; 7 Department of Translational Immunology, Genentech Inc., 1 DNA Way, South San Francisco, CA 94084, USA

**Keywords:** hypoxia, high altitude, high-altitude pulmonary edema, pulmonary hypertension, inflammation, immune cells, classical monocytes

## Abstract

High-altitude pulmonary edema (HAPE) is a rapidly progressive, life-threatening disorder arising in otherwise healthy individuals upon ascent to high altitude, yet the mechanisms underlying maladaptive vascular leak remain poorly defined. Although elevated pulmonary arterial pressure and capillary stress failure are recognized as central hemodynamic drivers, accumulating evidence indicates that innate immune dysregulation is an equally critical, largely unexplored determinant of HAPE. To systematically delineate the immune and molecular programs that distinguish pathological responses to hypobaric hypoxia from acclimatization, peripheral blood along with clinical details was collected from low-altitude controls (LA-Cntrl, number of participants, (n = 19), healthy high-altitude sojourners (HA-Cntrl, n = 47), and HAPE patients (n = 90). Plasma proteomic markers were quantified using a targeted panel, while monocyte and dendritic cell subsets in peripheral blood mononuclear cells were immunophenotyped by multicolor flow cytometry. HA-Cntrl subjects displayed an anti-inflammatory profile, marked by the suppression of CXC chemokine receptor 3 axis chemokines. HAPE patients, in contrast, exhibited a pro-inflammatory, vascular injury signature, with elevated levels of inflammatory interleukins and myeloid and chemotactic factors. This inflammatory signature was accompanied by the expansion of classical monocytes, implicating a myeloid vascular program associated with HAPE.

## 1. Introduction

High-altitude pulmonary edema (HAPE) is a life-threatening form of acute altitude illness that occurs in otherwise healthy individuals after rapid ascent to high altitude (HA) [1,2]. It has classically been attributed to exaggerated hypoxic pulmonary vasoconstriction, elevated pulmonary arterial pressure, and stress failure of the pulmonary capillary barrier, leading to alveolar fluid accumulation [1,2]. While this hemodynamic framework explains key features of disease physiology, it does not fully account for inter-individual variability in susceptibility, severity, or progression. Efforts to define the molecular basis of HAPE have been constrained by the remote locations of high-altitude environments and by the lack of robust experimental models that recapitulate the human phenotype [3]. Consequently, the cellular mechanisms linking hypoxic exposure to endothelial dysfunction and vascular leaks remain incompletely understood, particularly with respect to the role of the immune system.

At high altitude, reduced oxygen availability resulting from hypobaric hypoxia inhibits prolyl hydroxylase activity, thereby stabilizing hypoxia-inducible factor 1-alpha (HIF-1α). Hypoxia also promotes the activation of nuclear factor kappa-light-chain-enhancer of activated B cells (NF-κB) and its translocation into the nucleus, where it can interact with HIF-1α [4,5]. NF-κB has also been reported to bind to HIF-1α, thereby constituting an important mechanistic link between hypoxic stress and inflammatory activation, promoting the transcription of pro-inflammatory cytokines, including interleukin-6 (IL-6), interleukin-1 beta (IL-1β), and tumor necrosis factor-alpha (TNF-α) [6,7]. Hypobaric hypoxia exposure has been shown to induce systemic inflammatory responses, elevate circulating cytokines and chemokines, and reshape immune cell composition across multiple altitude-associated disorders, including acute mountain sickness, high-altitude pulmonary hypertension (HAPH), HAPE, and high-altitude cerebral edema, positioning immune regulation as a critical determinant of high-altitude adaptation and disease susceptibility [8,9,10]. One such study has demonstrated that hypoxia activates innate immune pathways, including HIF-1α- and NLRP3-mediated signaling, resulting in enhanced production of inflammatory mediators, endothelial activation, vascular remodeling, and thromboinflammatory responses [11].

HIF-1α also acts as a master regulator of immune cell metabolism, pushing myeloid cells toward anaerobic glycolysis, a shift essential for their survival and function under hypoxic stress [12], and playing important roles in the immune system, including physical defense and roles in both innate and acquired immunity [13]. Therefore, myeloid cells, such as monocytes and macrophages among immune populations, have emerged as important mediators of hypoxia-induced pathology [14,15]. In experimental pulmonary hypertension, hypoxia induces expansion of interstitial macrophages and recruitment of C-C motif chemokine receptor 2 (CCR2^+^) monocyte-derived cells through C-C motif chemokine ligand 2 (CCL2) dependent signaling, contributing directly to vascular remodeling and disease progression [16]. Likewise, genetic and pharmacological enhancement of PHD2 activity has been shown to suppress hypoxia-induced monocyte activation and reduce IL-6 and IL-1β production, highlighting the importance of myeloid cell regulation in determining adaptation outcomes under low-oxygen conditions [14]. Consistent with this concept, our recent multi-omics analysis of HAPE identified coordinated activation of innate immune and hypoxia-responsive networks in peripheral blood [17]. The study integrated transcriptomic and cytokine profiling, revealing the enrichment of inflammatory and endothelial dysfunction pathways, including oncostatin-M (OSM)- and NF-κB-associated signaling modules, as well as regulatory network changes linked to leukocyte activation and vascular remodeling.

Together, these observations hypothesize that HAPE development is associated with immune alterations that extend beyond generalized hypoxic effects and reflect distinct cellular programs underlying maladaptive responses. To investigate this, we first performed plasma cytokine profiling across healthy lowland controls (LA-Cntrl), who were never exposed to HA; hypoxia-tolerant individuals who remained free of any HA illnesses, including HAPE, despite HA exposure (HA-Cntrl); and patients with established HAPE to identify inflammatory cytokines specifically associated with disease rather than hypoxic exposure alone. The resulting cytokine signatures provided the rationale for subsequent immune cell characterization, particularly of monocytes and dendritic cells, which have emerged as central regulators of hypoxia-induced inflammation, vascular remodeling, and immune homeostasis [18]. Multicolor flow cytometry was employed to comprehensively define circulating monocyte and dendritic cell subsets across the three study groups. By integrating cytokine profiling with cellular immune phenotyping, we sought to identify immune signatures that distinguish acclimatization to hypobaric hypoxia from the inflammatory responses associated with HAPE.

## 2. Results

### 2.1. Demographic and Clinical Characteristics of Study Subjects

**Figure 1:**
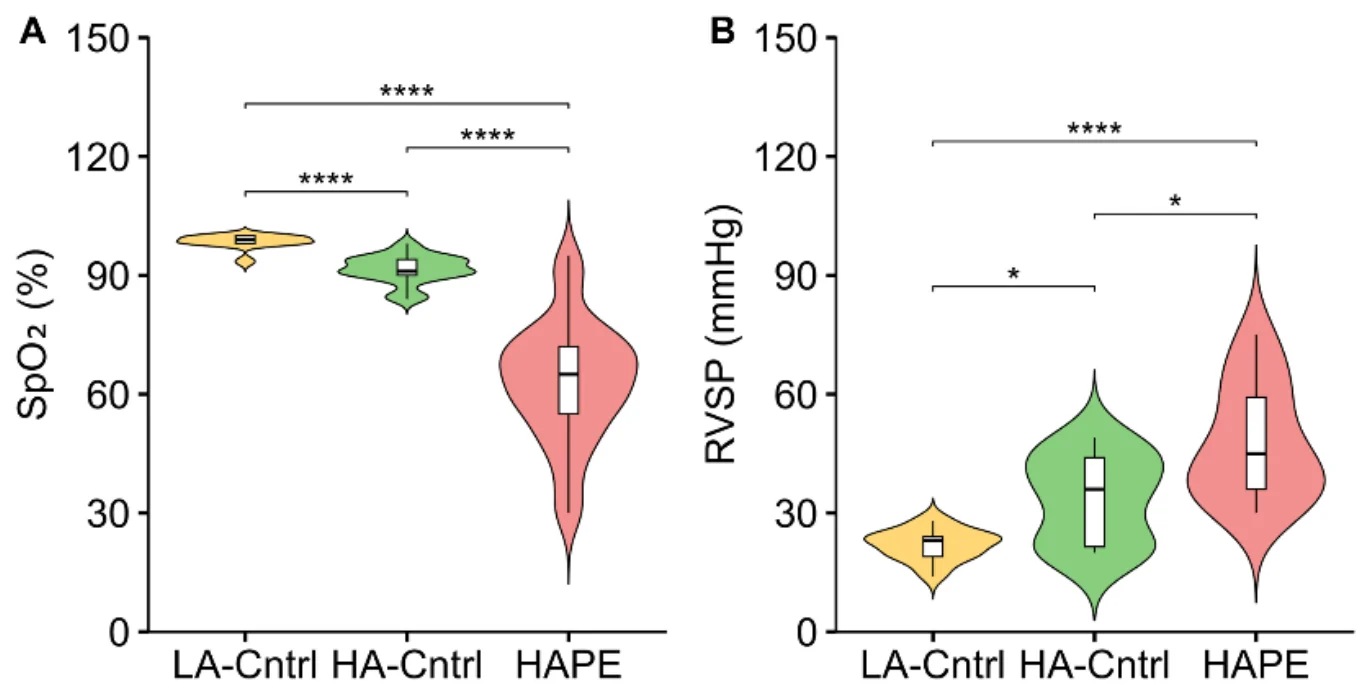
Peripheral oxygen saturation (SpO_2_) and right ventricular systolic pressure (RVSP) in the study groups. (**A**) Reduced SpO_2_ levels were observed in HAPE patients (n = 90) compared to HA-Cntrl (n = 47) and LA-Cntrl (n = 19). (**B**) RVSP levels were higher in HAPE patients (n = 15) compared to HA-Cntrl (n = 15) and LA-Cntrl (n = 17). HA-Cntrl: high altitude control; HAPE: high-altitude pulmonary edema patients; LA-Cntrl: low altitude control; RVSP: right ventricular systolic pressure; SpO_2_: peripheral oxygen saturation * *p* < 0.05 and **** *p* < 0.0001.

Demographic characteristics, including age and sex, as well as clinical parameters such as peripheral oxygen saturation (SpO_2_), respiratory rate (RR), pulse rate (PR), systolic blood pressure (SBP), diastolic blood pressure (DBP), and mean arterial pressure (MAP), are presented in Table 1. The first comparison, LA-Cntrl vs. HA-Cntrl, identified alterations associated with acclimatization to hypoxia by contrasting baseline LA controls with hypoxia-tolerant HA controls. The second comparison, HA-Cntrl vs. HAPE, characterized the signature specifically associated with HAPE in a case–control design by comparing hypoxia-tolerant HA controls with HAPE patients. Clinical assessment revealed significant physiological alterations in patients with HAPE (Table 1). SpO_2_ was markedly reduced in HAPE patients compared with the HA-Cntrl subjects (62.94 ± 15.15 vs. 91.48 ± 3.50; *p* < 0.0001, Figure 1A). In contrast, SpO_2_ did not differ between the HA-Cntrl and LA-Cntrl subjects (91.48 ± 3.50 vs. 98.86 ± 0.88; *p* = 0.5935, Figure 1A). Similarly, RR was significantly higher in HAPE patients than in the HA-Cntrl subjects (25.84 ± 4.58 vs. 19.46 ± 1.87; *p* < 0.0001), whereas no significant difference was observed between HA-Cntrl and LA-Cntrl subjects (19.46 ± 1.87 vs. 18.6 ± 1.30; *p* = 0.1064). Likewise, PR was significantly elevated in HAPE patients compared with HA-Cntrl subjects (108.9 ± 21.30 vs. 85.55 ± 13.43; *p* < 0.0001) but did not differ between HA-Cntrl and LA-Cntrl subjects (85.55 ± 13.43 vs. 86.0 ± 7.40; *p* = 0.8899). HAPE patients exhibited significantly higher MAP than the HA-Cntrl subjects (97.10 ± 12.92 vs. 92.77 ± 6.93; *p* < 0.05), as well as between HA-Cntrl and LA-Cntrl subjects (92.77 ± 6.93 vs. 99.2 ± 14.54; *p* < 0.05). Right ventricular systolic pressure (RVSP) measured through echocardiography differed significantly between the three groups, with a significant increase in HAPE patients (Figure 1B).

### 2.2. Targeted Plasma Proteome Profiling Revealed Inflammatory Signatures in HAPE Patients

**Figure 2:**
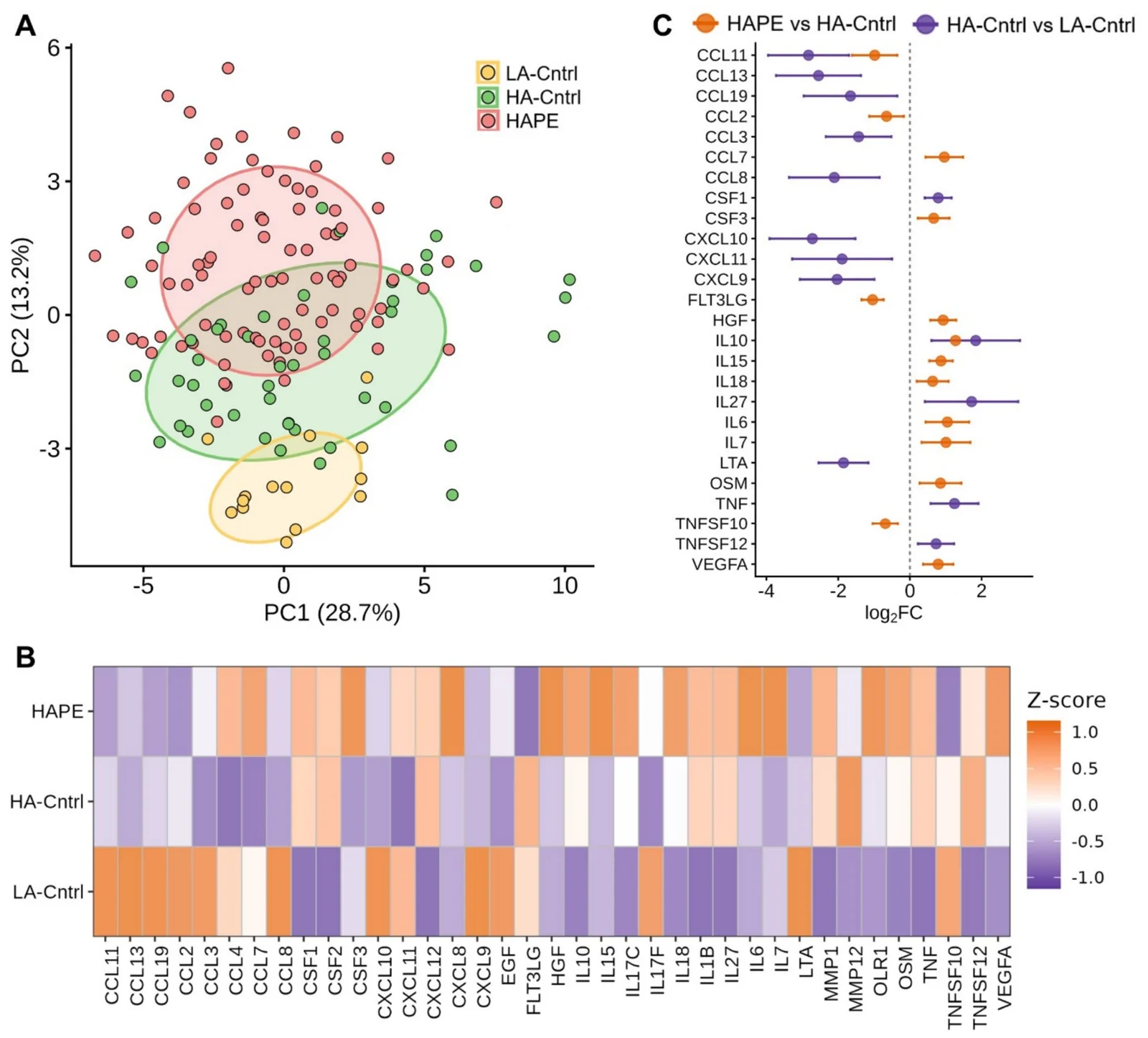
Plasma proteomic profiling reveals distinct signatures in HAPE patients. (**A**) Principal component analysis (PCA) of Olink plasma proteomic data showing separation among LA-Cntrl, HA-Cntrl, and HAPE groups, indicating distinct proteomic landscapes across the three cohorts. (**B**) Heatmap of QC-passed cytokines showing Z-score-normalized mean expression values across groups. The color scale represents relative expression level, ranging from purple (low) to orange (high). (**C**) Forest plot of cytokines significantly altered in at least one pairwise comparison. Cytokines on the right side indicate higher expression, and those on the left indicate lower expression in the respective comparison. Orange bars represent the HAPE vs. HA-Cntrl comparison, and purple bars represent the HA-Cntrl vs. LA-Cntrl comparison. HA-Cntrl: high altitude control; HAPE: high-altitude pulmonary edema patients; LA-Cntrl: low altitude control.

**Figure 3:**
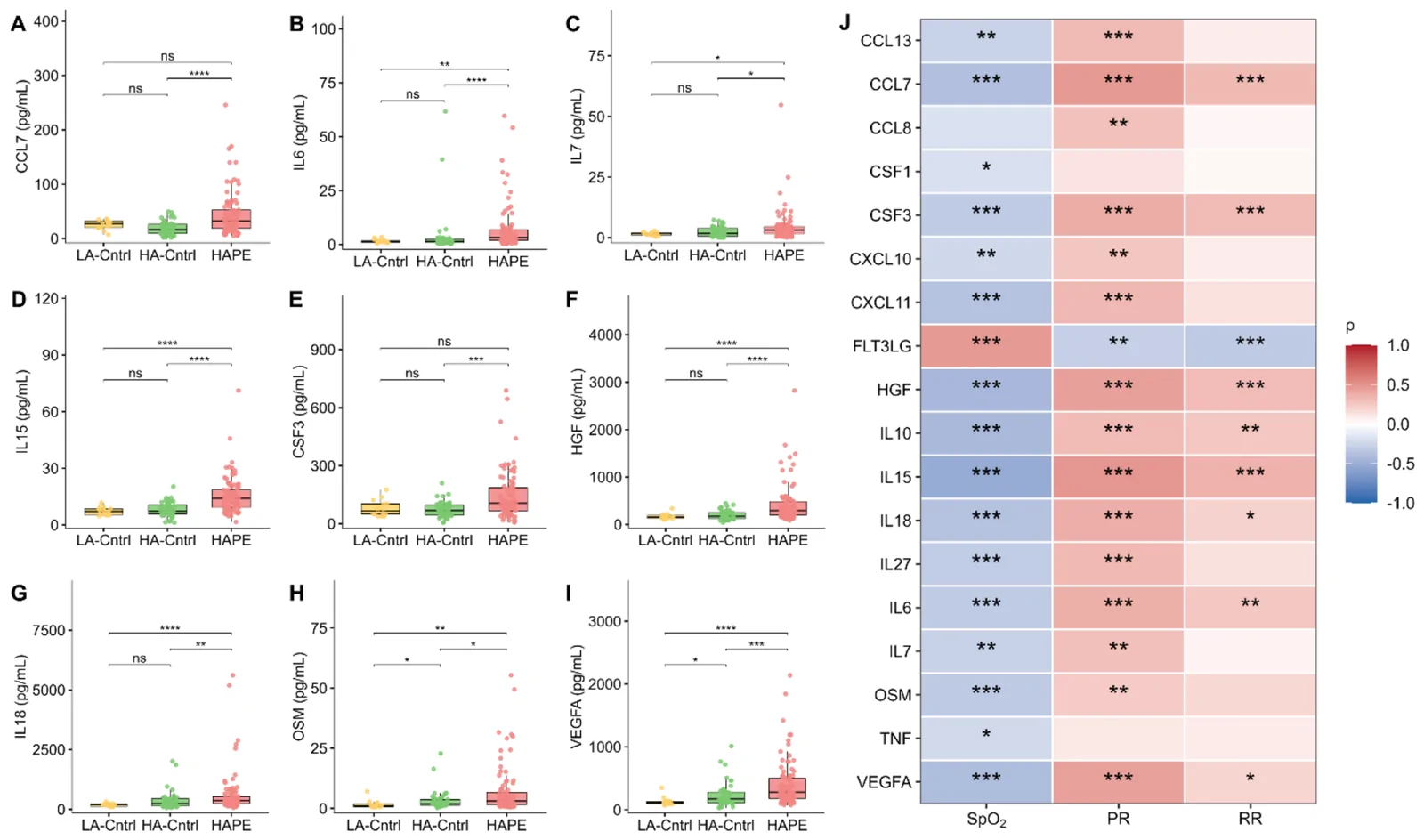
Differential plasma proteome profiles and their correlation with clinical parameters. (**A**–**I**) Boxplots depicting absolute quantification (pg/mL) of cytokines uniquely dysregulated in HAPE compared to HA-Cntrl. (**J**) Heatmap displaying Spearman rank correlation coefficients (ρ) between clinical variables, oxygen saturation (SpO_2_), respiratory rate (RR), and pulse rate (PR), and cytokines in hypoxia-exposed groups. The color scale ranges from blue (negative correlation) to red (positive correlation), with white indicating no correlation. * *p* < 0.05, ** *p* < 0.01, *** *p* < 0.001, and **** *p* < 0.0001, ns: not significant.

Plasma proteomic profiling was performed on samples from three study groups: LA-Cntrl, HA-Cntrl, and HAPE patients. Principal component analysis (PCA) after batch corrections effectively distinguished the three groups, with PC1 and PC2 accounting for 28.7% and 13.2% of the total variance, respectively (Figure 2A), indicating distinct plasma proteomic signatures associated with altitude exposure and HAPE status. The heatmap of group-wise mean expression of 37 cytokines that passed quality control revealed distinct patterns among the three groups (Figure 2B). Pairwise comparisons identified 26 of the 37 cytokines as significantly altered in at least one comparison, as depicted in the forest plot of log2 fold change (log2FC) (Figure 2C). In HAPE patients relative to HA-Cntrl subjects, C-C motif chemokine ligand 7 (CCL7), colony stimulating factor 3 (CSF3), hepatocyte growth factor (HGF), interleukin-15 (IL-15), interleukin-18 (IL-18), IL-6, interleukin 7 (IL-7), oncostatin M (OSM), and vascular endothelial growth factor (VEGFA) were significantly elevated, whereas C-C motif chemokine ligand 2 (CCL2), Fms-related tyrosine kinase 3 ligand (FLT3LG), and tumor necrosis factor ligand superfamily member 10 (TNFSF10) were significantly lower (Figure 2C). In HA-Cntrl subjects relative to LA-Cntrl subjects, colony stimulating factor 1 (CSF1), interleukin 27 (IL-27), tumor necrosis factor (TNF), and tumor necrosis factor ligand superfamily member 12 (TNFSF12) were significantly elevated, whereas C-C motif chemokine ligand 13 (CCL13), C-C motif chemokine ligand 19 (CCL19), C-C motif chemokine ligand 3 (CCL3), C-C motif chemokine ligand 8 (CCL8), C-X-C motif chemokine ligand 10 (CXCL10), C-X-C motif chemokine ligand 11 (CXCL11), C-X-C motif chemokine ligand 9 (CXCL9), and lymphotoxin-alpha (LTA) were significantly lower (Figure 2C). C-C motif chemokine ligand 11 (CCL11) and interleukin-10 (IL-10) were the two cytokines common to both comparisons, with CCL11 consistently lower and IL-10 consistently elevated across both (Figure 2C). Further, Figure 3A–I present the absolute quantification levels of CCL7, IL-6, IL-7, IL-15, CSF3, HGF, IL-18, OSM, and VEGFA that were uniquely and significantly elevated in HAPE patients compared to HA-Cntrl subjects.

### 2.3. Correlation of Differential Plasma Proteome Profiles with Clinical Parameters

Spearman’s rank correlation analysis was performed to evaluate the association between plasma cytokine levels and the clinical parameters SpO_2_, RR, and PR in the hypoxia-exposed groups (Figure 3J). The analysis revealed predominantly negative correlations between cytokine expression and SpO_2_ levels. The cytokines most strongly and consistently inversely associated with SpO_2_ were CCL13 (ρ = −0.27, *p* < 0.01), CCL7 (ρ = −0.39, *p* < 0.001), CSF1 (ρ = −0.19, *p* < 0.05), CSF3 (ρ = −0.32, *p* < 0.001), CXCL10 (ρ = −0.23, *p* < 0.01), CXCL11 (ρ = −0.37, *p* < 0.001), HGF (ρ = −0.44, *p* < 0.001), IL-10 (ρ = −0.43, *p* <0.001), IL-15 (ρ = −0.52, *p* <0.001), IL-18 (ρ = −0.37, *p* < 0.001), IL-6 (ρ = −0.32, *p* < 0.001), IL-7 (ρ = −0.28, *p* < 0.01), OSM (ρ = −0.32, *p* < 0.001), TNF (ρ = −0.23, *p* < 0.05), and VEGFA (ρ = −0.41, *p* < 0.001) (Figure 3J). In contrast, positive correlations were observed between cytokine levels and both RR and PR. PR demonstrated the broadest pattern of associations, with significant positive correlations identified for CCL13 (ρ = 0.32, *p* < 0.001), CCL7 (ρ = 0.47, *p* < 0.001), CCL8 (ρ = 0.28, *p*< 0.01), CSF3 (ρ = 0.38, *p* < 0.001), CXCL10 (ρ = 0.26, *p* < 0.01), CXCL11 (ρ = 0.33, *p* < 0.001), HGF (ρ = 0.44, *p* < 0.001), IL-10 (ρ = 0.31, *p* < 0.001), IL-15 (ρ = 0.48, *p* < 0.001), IL-18 (ρ = 0.37, *p* < 0.001), IL-27 (ρ = 0.32, *p* < 0.001), IL-6 (ρ = 0.36, *p* < 0.001), IL-7 (ρ = 0.29, *p* < 0.01), OSM (ρ = 0.24, *p* < 0.01), and VEGFA (ρ = 0.43, *p*< 0.001) (Figure 3J). Similarly, RR showed positive associations with CCL7 (ρ = 0.32, *p* < 0.001), CSF3 (ρ = 0.31, *p* < 0.001), HGF (ρ = 0.31, *p* < 0.001), IL-10 (ρ = 0.26, *p* < 0.01), IL-15 (ρ = 0.35, *p* < 0.001), IL-18 (ρ = 0.21, *p* < 0.05), IL-6 (ρ = 0.26, *p* < 0.01), and VEGFA (ρ = 0.19, *p* < 0.05) (Figure 3J). Notably, FLT3LG exhibited a divergent pattern, showing a positive association with SpO_2_ (ρ = 0.47, *p* < 0.001) but inverse associations with both PR (ρ = −0.29, *p* <0.01) and RR (ρ = −0.32, *p* < 0.001), distinguishing it from the broader inflammatory signature.

### 2.4. Distinct Monocyte Immunophenotypes Associated with HAPE Pathophysiology

**Figure 4:**
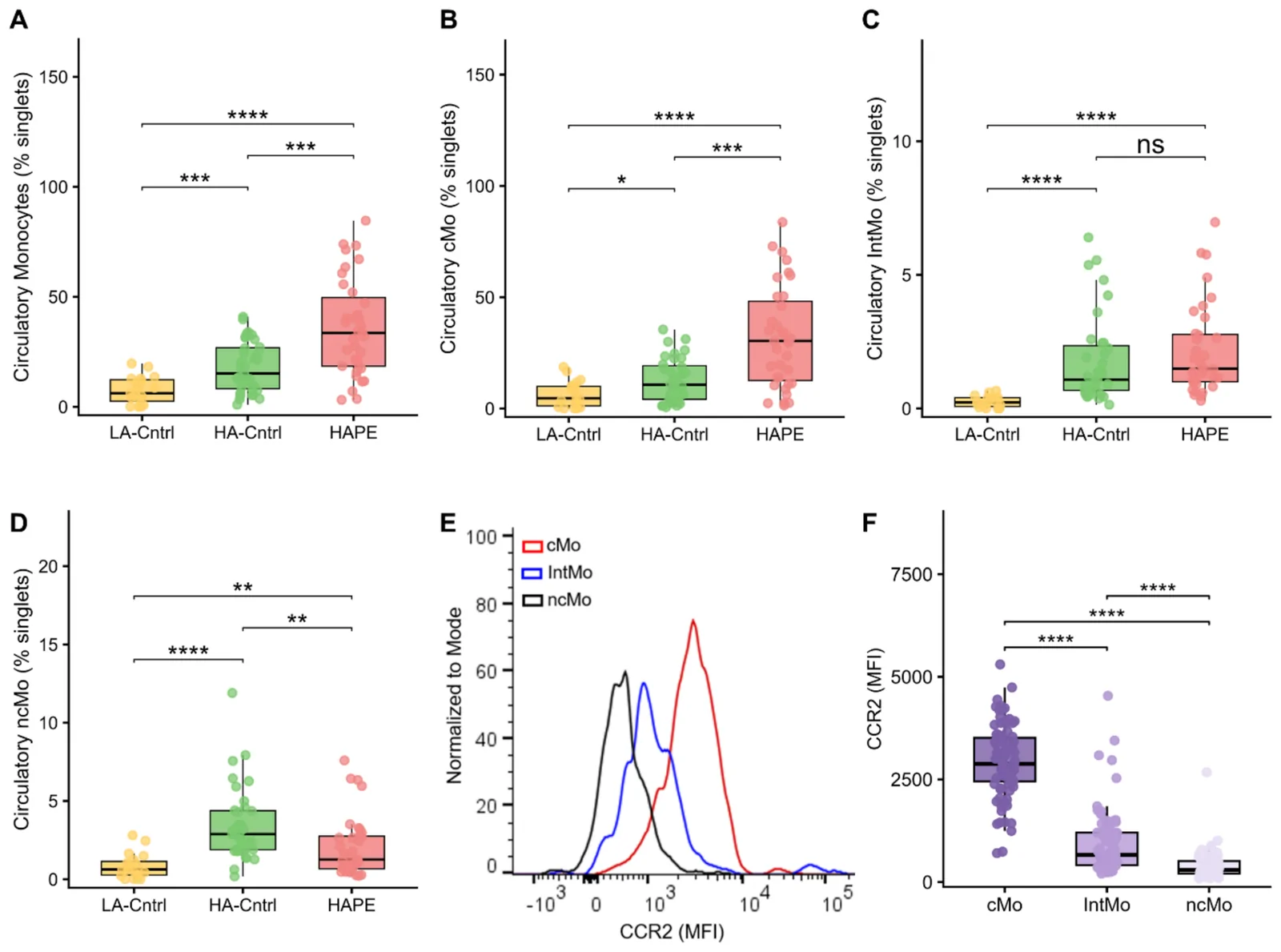
Quantification of intravascular monocytes in HAPE. (**A**) Circulatory monocytes in the study groups. (**B**) Higher circulatory classical monocytes in HAPE patients. (**C**) Circulatory intermediate monocytes in the study groups. (**D**) Blunted circulatory non-classical monocytes in HAPE patients. (**E**,**F**) Mean fluorescence intensity of CCR2 in the monocyte subsets. CCR2: C-C motif chemokine receptor 2; cMo: classical monocytes; HA-Cntrl: high altitude control; HAPE: high-altitude pulmonary edema patients; IntMo: intermediate monocytes; LA-Cntrl: low altitude control; ncMo: non classical monocytes. * *p* < 0.05, ** *p* < 0.01, *** *p* < 0.001, and **** *p* < 0.0001, ns: not significant.

Flow cytometry-based quantitative analysis of peripheral blood mononuclear cells was performed in LA-Cntrl, HA-Cntrl, and HAPE patients (Figure S1). The gating strategy utilized for characterization of intravascular monocytes and their subsets is shown in Figure S2. Circulatory monocytes were significantly increased in HAPE patients compared to HA-Cntrl subjects, and in HA-Cntrl subjects compared to LA-Cntrl subjects (Figure 4A). Further characterization revealed a significantly elevated proportion of CD14^+^CD16^−^ classical monocytes (cMo) in HAPE patients compared to HA-Cntrl subjects and in HA-Cntrl subjects compared to LA-Cntrl subjects (Figure 4B). However, CD14^+^CD16^+^ intermediate monocytes (IntMo) were not significantly different in HAPE patients compared to HA-Cntrl, but they were significantly higher in HA-Cntrl compared to LA-Cntrl (Figure 4C). In contrast, CD14^−^CD16^+^ non-classical monocytes (ncMo) were significantly reduced in HAPE patients and increased in HA-Cntrl subjects compared to LA-Cntrl subjects (Figure 4D). To further validate the monocyte subset, we assessed CCR2expression, a characteristic marker of classical monocytes (cMo). The median fluorescence intensity (MFI) of CCR2 was markedly higher in cMo compared to IntMo and ncMo (Figure 4E,F).

### 2.5. Dendritic Cell Subsets in HAPE

As circulating dendritic cells (DCs) are key antigen-presenting cells that bridge innate and adaptive immunity and can reflect systemic immune activation, we next assessed their frequencies in peripheral blood. The gating strategy utilized for the characterization of intravascular DCs and their subsets is shown in Figure S2. There was no significant difference in circulatory DC levels in HAPE patients compared to HA-Cntrl, and in HA-Cntrl subjects compared to LA-Cntrl subjects (Figure S3A). Similarly, no significant difference was observed in conventional type 1 dendritic cells (cDC1) and conventional type 2 dendritic cells (cDC2) subsets of DCs in HAPE patients compared to HA-Cntrl, and in HA-Cntrl subjects compared to LA-Cntrl subjects (Figure S3B,C).

## 3. Discussion

The present study combined targeted plasma cytokine profiling with detailed immune cell phenotyping across three groups, namely sea-level controls (LA-Cntrl), who were never exposed to HA, and two HA-exposed sojourner groups, those who acclimatized without adverse incidents (HA-Cntrl) and those who failed to acclimatize (HAPE patients), to identify the circulating cytokines and cellular signatures that separate acclimatization from HAPE. Our findings from the two comparison groups reveal distinct signatures. A comparison of HA-Cntrl with LA-Cntrl reveals the immune program associated with successful acclimatization to high altitude, whereas the comparison of HA-Cntrl with HAPE identifies immune signatures associated with the failure to maintain this adaptive response, resulting in HAPE.

Relative to LA-Cntrl, HA-Cntrl subjects exhibited lower circulating levels of CCL3, CCL8, CCL11, CCL13, CCL19, CXCL9, CXCL10, CXCL11, and LTA, together with higher levels of CSF1, IL-10, IL-27, TNF, and TNFSF12. Notably, the suppressed chemokine profile was dominated by the CXCR3 ligands CXCL9, CXCL10, and CXCL11, indicating a role for IFN-γ-associated inflammatory signaling [19,20]. This dampened inflammatory state was accompanied by the induction of a regulatory module centered on IL-10 and IL-27, both of which promote immune homeostasis by suppressing excessive inflammatory responses [21,22]. This response was further supported by CSF1, which promotes monocyte survival and reparative macrophage differentiation [23], and TNFSF12, which modulates endothelial repair and vascular remodeling [24]. Together, these findings suggest that successful acclimatization is not a state of immune quiescence but rather an actively regulated immune program characterized by IFN-γ-associated inflammatory signaling and recruitment of inflammatory cells, coupled with enhanced immunoregulatory and tissue-adaptive responses.

Despite the established role of inflammatory immune cells in diverse pulmonary diseases [25], the immune alterations that drive high-altitude pulmonary edema (HAPE) remain insufficiently understood. HAPE has been viewed as a predominantly hemodynamic disorder initiated by exaggerated hypoxic pulmonary vasoconstriction, resulting in elevated pulmonary arterial pressure, overperfusion, and stress failure of the alveolar–capillary barrier. The inflammatory responses have generally been interpreted as secondary consequences of vascular injury rather than drivers of the disease [26]. However, growing evidence challenges this view, demonstrating that hypobaric hypoxia actively modulates innate immune responses [27], promoting leukocyte activation and trafficking [28] and inducing endothelial dysfunction [29].

The present study observed elevated levels of CCL7, CSF3, HGF, IL-6, IL-7, IL-15, IL-18, OSM, and VEGFA, together with reduced CCL2, CCL11, FLT3LG, and TNFSF10 in HAPE when compared with HA-Cntrl. Collectively, these changes suggest coordinated activation of pathways involved in monocyte recruitment (CCL7), myelopoiesis (CSF3 and IL-6), endothelial activation (IL-6, IL-18, IL-15, and OSM), and vascular remodeling and permeability (VEGFA and HGF). Elevated levels of CCL7, a key chemokine that promotes the mobilization of CCR2^+^ monocytes from the bone marrow, likely drive their accumulation in circulation [30]. These cells can then infiltrate inflamed lung tissue and differentiate into inflammatory CCR2^+^ interstitial macrophages, a process supported by increased CSF3 expression, which promotes monocyte maturation and survival [31]. Elevated IL-7 and IL-15 likely represent compensatory homeostatic responses to lymphocyte stress [32], whereas increased IL-18 is consistent with sustained innate immune activation [33]. The elevated pro-inflammatory cytokine profile was dominated by IL-6, which induces the production of VEGFA and HGF, thereby promoting angiogenesis and increasing vascular permeability, two hallmark features of HAPE [34,35,36]. OSM, another member of the IL-6 cytokine family, regulates endothelial barrier integrity and extracellular matrix remodeling through JAK/STAT, MAPK, and PI3K-AKT signaling, consistent with our previous findings [17]. Elevated HGF likely reflects a compensatory reparative response, given its established role in protecting epithelial and endothelial barriers and promoting tissue repair following vascular injury [37]. Supporting the clinical relevance of these pathways, circulating cytokine levels correlated with physiological markers of illness, including oxygen saturation, pulse rate, and respiratory rate.

**Figure 5:**
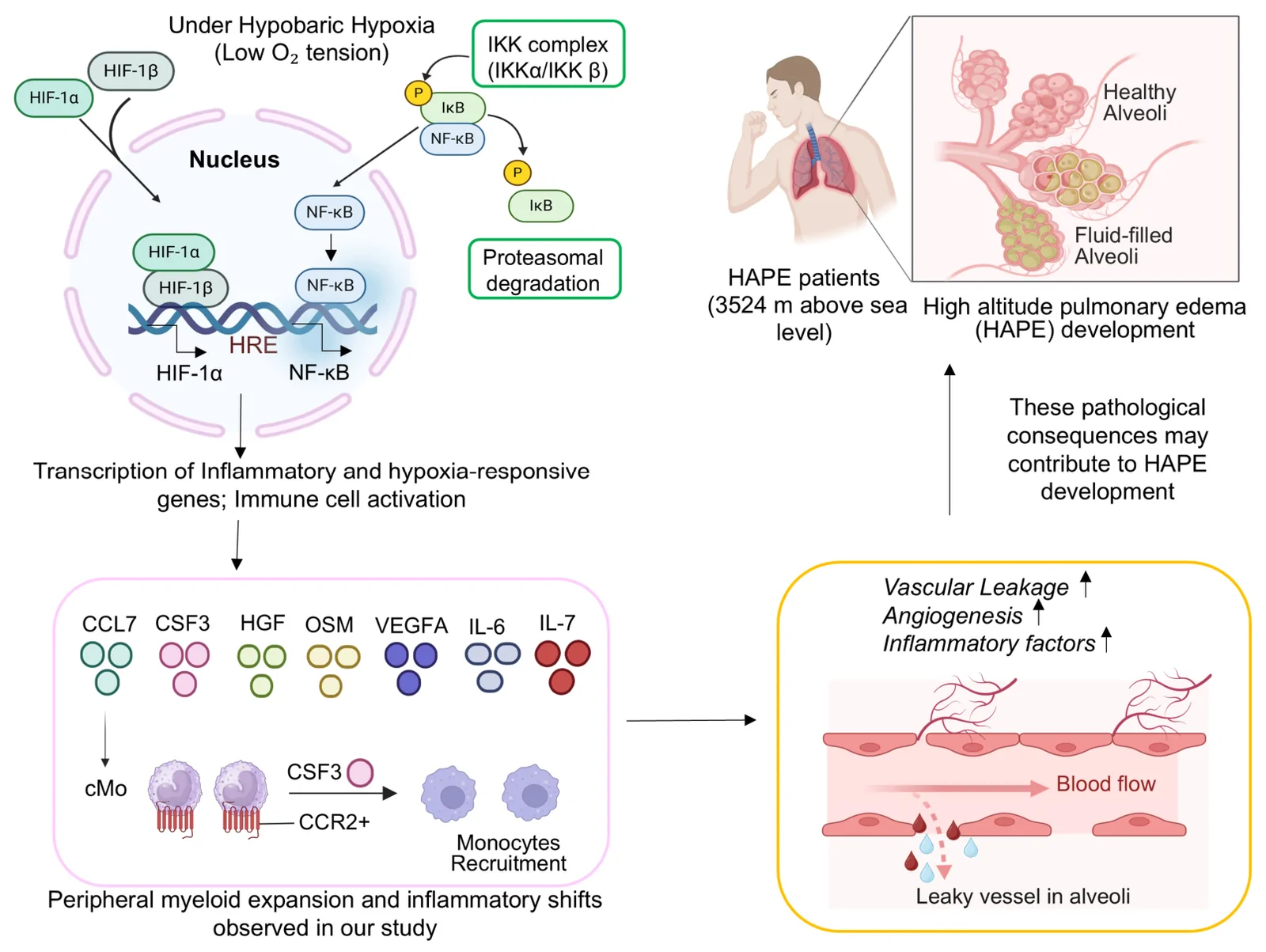
Schematic illustration of the molecular cascade linking hypobaric hypoxia exposure to peripheral myeloid expansion and inflammatory alterations observed in our study. CCL7: C-C motif chemokine ligand 7; CCR2: C-C motif chemokine receptor 2; cMo: classical monocytes; CSF3: colony stimulating factor 3; HGF: hepatocyte growth factor; HIF-1α: hypoxia-inducible factor 1-alpha; HIF-1β: hypoxia-inducible factor 1-beta; HRE: hypoxia response element; IκB: inhibitor of nuclear factor kappa B; IKKα: inhibitor of nuclear factor kappa-B kinase subunit alpha; IKKβ: inhibitor of nuclear factor kappa-B kinase subunit beta; IL-6: interleukin 6; IL-7: interleukin 7; NF-κB: nuclear factor kappa-light-chain-enhancer of activated B cells; OSM: oncostatin M; VEGFA: vascular endothelial growth factor A.

Previous studies have evaluated either circulating inflammatory mediators or immune cell alterations in isolation, leaving the relationship between cytokine dysregulation and changes in immune cell composition largely unexplored, further limiting the understanding of how systemic immune activation is associated with HAPE pathophysiology. The cytokine signature suggested activation of the myeloid lineage cells, prompting a closer look at circulatory monocyte and dendritic cell subsets. Bone marrow-derived monocytes are conventionally divided by CD14 and CD16 expression into classical (cMo: CD14^+^CD16^−^), intermediate (IntMo: CD14^+^CD16^+^), and non-classical (ncMo: CD14^−^CD16^+^) populations [38,39,40]. Similarly, the bone marrow-derived dendritic cells [41] divide into CD103^+^ (cDC1) and CD103^−^ (cDC2) subsets [42]. In line with elevated CCL7, a CCR2 ligand that governs monocyte mobilization from the bone marrow and recruitment to inflamed tissues, HAPE patients showed expansion of total classical monocytes, with an increase in CCR2^+^ classical monocytes. Previous studies have shown that the same subset contributes to pulmonary hypertension under hypoxic conditions [16,43]. More broadly, CCR2^+^ monocytes and interstitial macrophages have been linked to arterial wall inflammation, pulmonary fibrosis, and elevated cardiopulmonary risk [44,45,46,47]. In experimental hypoxic PH models, CCR2^+^ cMo are recruited to the lung specifically via CCL2 produced by resident interstitial macrophages [16]. The expansion of circulating CCR2^+^ classical monocytes observed in our cohort likely represents the systemic counterpart to the localized inflammatory events well characterized in pulmonary tissue during HAPE pathogenesis. Previous bronchoalveolar lavage fluid (BALF) studies in established HAPE have demonstrated marked infiltration of alveolar macrophages and neutrophils, along with elevated pro-inflammatory cytokines, including IL-1β, IL-6, IL-8, and TNF-α [48,49]. Conversely, BAL performed within the initial hours of high-altitude ascent, prior to the onset of full clinical symptoms, reveals protein-rich fluid and erythrocyte leakage across the alveolar space in the absence of overt leukocytosis [50]. This suggests that mechanical stress and capillary barrier failure precede secondary leukocyte recruitment. Taken together with our observed shifts in peripheral myeloid subsets and the established CCL2–CCR2 signaling axis, these findings support a temporal cascade wherein exaggerated hypoxic pulmonary vasoconstriction compromises the alveolar–capillary barrier. In response to local shear stress and tissue micro-injury, resident pulmonary cells release CCL2, actively recruiting expanded circulating CCR2^+^ classical monocytes across the compromised barrier into the alveolar space, where they exacerbate microvascular permeability and tissue inflammation. The corresponding drop in circulating non-classical monocytes (ncMo) is notable, since murine hypoxic PH models show ncMo entering the lung and differentiating into interstitial macrophages [51], suggesting that the ncMo decrease and cMo increase seen here may reflect redistribution toward the lung, through greater marrow output, pulmonary recruitment, or both, rather than an overall change in monocyte production. The lack of a validated HAPE animal model and the inaccessibility of human lung tissue currently keep this at the level of a mechanistic hypothesis. Additionally, our results showed a decrease in CCL2, which is mostly tissue-specific and less available in circulation, and an increase in CCL7, which is mostly circulatory and an equally effective driver of inflammatory monocyte recruitment during tissue injury [52]. Although circulating FLT3LG levels were reduced in HAPE, this was not accompanied by significant changes in the circulating dendritic cell subsets. In addition, reduced CCL11 indicates a suppressed eosinophilic component in HAPE, further supporting a myeloid, CCR2-centered inflammatory profile. Lower TNFSF10 levels suggest slow inflammatory resolution, given its role in clearing activated immune cells via apoptosis [53]. Thus, our findings can be interpreted in the context of a previously established molecular response to severe hypobaric hypoxia (Figure 5). Low tissue *p*O2 inhibits prolyl hydroxylases, stabilizing HIF-1α and driving a metabolic switch toward anaerobic glycolysis in circulating myeloid cells [12]. Previous studies from our group and others have further demonstrated that HIF stabilization and its crosstalk with inflammatory signaling pathways can promote monocyte survival and effector functions, contributing to a pro-inflammatory cytokine milieu [6,7,16]. Building on these established upstream molecular mechanisms, our current study provides evidence at the cellular and inflammatory levels, demonstrating alterations in peripheral myeloid populations, immune cell activation, and cytokine profiles following severe hypobaric hypoxia. Together, these findings provide a mechanistic framework linking the previously described hypoxia-responsive molecular cascade to the peripheral myeloid expansion and inflammatory changes observed in our study (Figure 5).

Several limitations should be acknowledged. Immune phenotyping was performed using peripheral blood mononuclear cells and therefore may not fully reflect immune events occurring within the pulmonary vasculature or alveolar compartment, where HAPE pathology develops. The targeted cytokine panel, while providing robust quantification of selected inflammatory mediators, does not capture the full complexity of the circulating proteome. Furthermore, the individuals in the HAPE cohort were, on average, older than those in the LA-Cntrl and HA-Cntrl groups. As a result, age was included as one of the covariates in all subsequent analyses. Finally, the cross-sectional nature of this study limits mechanistic inference regarding temporal relationships between cytokine alterations, immune cell remodeling, and disease progression. Future longitudinal studies integrating peripheral and pulmonary immune profiling will be essential to establish causal pathways linking hypoxic exposure to immune dysregulation and vascular injury.

## 4. Materials and Methods

### 4.1. Study Groups

This study included 156 individuals of Indo-Aryan ethnicity recruited in collaboration with the CSIR-IGIB, Delhi, India, and the Sonam Norboo Memorial (SNM) Hospital, Leh, Ladakh, India. Our study included male and female patients across all study groups, and sex was treated as a biological variable in the analysis.

The participants were divided into three groups comprising (1) healthy low-altitude controls (LA-Cntrl, N = 19), who lived at lowland altitudes and were never exposed to HA environments, (2) healthy HA controls (HA-Cntrl, N = 47), who were lowlanders who went to HA in Ladakh, India, and did not develop any HA illnesses, including HAPE, and (3) HAPE patients (HAPE, N = 90), who were sojourners who went to HA in a similar condition to HA-Cntrl individuals but were diagnosed with HAPE within 2–5 days of arrival by physicians at SNM Hospital. Clinically, HAPE patients presented with chest tightness or pain, persistent cough, and dyspnea at rest. Physical examination revealed pulmonary rales, cyanosis, tachycardia, and tachypnea, accompanied by reduced SpO_2_ levels indicative of hypoxemia. Chest radiography demonstrated infiltrates consistent with pulmonary edema [54]. Individuals in whom hypoxemia could be attributed to alternate causes, such as pneumonia, were excluded following clinical assessment. A comprehensive questionnaire was administered to all participants, capturing information on medical history, pre-existing conditions, time elapsed since diagnosis, and the use of standard prophylactic agents for HA illness prior to ascent. Participants with known thyroid, hepatic, hematological, or cardiovascular conditions were excluded from the study, as were those with hypoxemia attributable to pneumonia or other respiratory disorders. Both the HA-Cntrl and HAPE participants travelled via flights to Leh, Ladakh, India (3524 m above sea level). Lake Louise scoring was applied to rule out acute mountain sickness in healthy controls [55]. Blood samples from HAPE patients were collected immediately following diagnosis, prior to the initiation of any therapeutic intervention, including supplemental oxygen administration. HA-Cntrl participants remained asymptomatic throughout and were sampled within the same 2–5-day window following arrival as the HAPE group, ensuring matched exposure durations across HA-Cntrl and HAPE participants.

### 4.2. Inclusion and Exclusion Criteria

Healthy volunteers aged 18–65 years were recruited with informed consent for the LA-Cntrl and HA-Cntrl groups. Exclusion criteria included chronic diseases, pregnancy, or inability to provide informed consent. Inclusion criteria for the HAPE group required participants to be aged 18–65 years and to have no history of chronic respiratory or cardiovascular disease.

### 4.3. Clinical Evaluation

Clinical parameters, including SpO_2_, RR, PR, SBP, DBP, and RVSP, were assessed. SpO_2_ was determined using a pulse oximeter (Criticare Systems Inc., Warwick, RI, USA). RVSP was estimated using continuous-wave Doppler echocardiography with an HP Sonos 5500 echocardiography system Hewlett-Packard, Andover, MA, USA) based on the modified Bernoulli equation, with right atrial pressure estimated from the size and collapsibility of the inferior vena cava [56]. Echocardiographic examinations were interpreted by local cardiologists. The assessed parameters included left and right ventricular size and function, tricuspid annular plane systolic excursion, tricuspid regurgitant velocity and pressure gradient across the tricuspid valve, and left and right atrial size.

### 4.4. Olink-Based High-Throughput Proteome Analysis

To assess concentrations of immune-related proteins, a specialized panel of 45 proteins, including interleukins and chemokines, was used. The assay used the proximity extension assay (PEA) technology on plasma samples, following the manufacturer’s instructions (Olink, Thermo Fisher, Waltham, MA, USA). In this assay, pairs of antibodies labeled with unique DNA oligonucleotides specifically bind to their target proteins in the plasma. Upon binding, the DNA tags are brought into close proximity, allowing them to hybridize and be extended by DNA polymerase, forming unique DNA sequences corresponding to each protein. These sequences are then amplified and quantified through real-time PCR in the Olink Signature Q100 system (Thermo Fisher, Waltham, MA, USA). Protein concentrations were calculated from standard curves and expressed as absolute values (pg/mL). Batch correction across runs was performed using the ComBat function from the sva package in R, and batch-corrected data were used for all subsequent visualization and statistical analyses. Differential protein expression analysis was performed using linear regression models implemented in the limma package in R, adjusting for age and sex as covariates. Effect sizes with an absolute log2 fold change of 1.5 and an associated adjusted *p*-value < 0.05, derived from this model, were used to generate the forest plots.

### 4.5. Peripheral Blood Mononuclear Cell (PBMC) Isolation

PBMCs were isolated from 6 mL of fresh blood collected directly into BD Vacutainer^®^ CPT™ Mononuclear Cell Preparation Tubes (BD Biosciences, Cat# 362753, San Jose, CA, USA). The tubes were centrifuged at 1500× *g* for 30 min at room temperature, according to the manufacturer’s recommended protocol. After centrifugation, the plasma and the PBMC-containing buffy coat were carefully collected into separate tubes. Plasma samples were stored at minus 80 °C. The PBMCs were transferred into 1 mL 1× PBS, then centrifuged at 400× *g* for 10 min at room temperature and resuspended in the freezing medium (10% DMSO + 90% FBS) before being cryopreserved at −80 °C in Leh, India. Subsequently, the samples were transported to the CSIR-IGIB in dry ice for downstream analysis.

### 4.6. Multicolor Flow Cytometry Analysis

For multicolor flow cytometry, cryopreserved PBMC samples from all three groups were thawed, and cell viability was assessed using the Trypan Blue exclusion method. The thawed cells were incubated with a fragment crystallizable (Fc) receptor blocker for 15 min to prevent nonspecific antibody binding. Without washing, the PBMCs were stained with monoclonal antibody (mAb) cocktails, including a panel of fluorophore-labeled antibodies targeting surface markers specific to monocytes and DC subsets, as detailed in Table S1. Total cells were first gated based on forward scatter (FSC-A) and side scatter (SSC-A), followed by exclusion of doublets using FSC-A versus FSC-H and FSC-A versus FSC-W plots to obtain singlets. This panel included markers for monocytes (CD14, CD16, and CCR2) and DCs (CD103 and cDC1). MHCII was included to identify antigen-presenting myeloid cells, while CD11b was used in combination with MHCII to define the myeloid compartment containing monocytes and conventional dendritic cells (DCs), enabling subsequent discrimination of these populations based on CD14, CD16, CD1c, and CD103 expression [41,57,58]. To exclude lineage-negative (Lin^−^) cells such as T cells, B cells, NK cells, and neutrophils, a “dump gate” was applied using a cocktail of antibodies targeting CD3, CD19, CDNK1.1, and Ly6G. Additionally, a viability dye was included to exclude dead cells. Data acquisition was performed using a BD FACSAria flow cytometer (BD Biosciences), and the acquired data were analyzed using FlowJo software (version 10.9.0).

### 4.7. Statistical Analysis

All statistical analyses and graphical representations were performed using R (version 4.3.2). Data normality was assessed using the Shapiro–Wilk test. Demographic and clinical variables were summarized as mean ± SD, while the Mann–Whitney U test was used to compare non-normally distributed data throughout the analysis. Multiple testing correction was performed using the Benjamini–Hochberg method to control the false discovery rate (FDR). A *p*-value threshold of less than 0.05 was considered statistically significant.

## 5. Conclusions

In conclusion, this study provides an integrated characterization of the cytokine signatures and circulating myeloid cell populations associated with HAPE. The findings indicate that hypobaric hypoxia induces an innate immune adaptation program in individuals who acclimatize successfully, combining restrained chemokine signaling with immunoregulation and reparative pathways. On the other hand, progression to HAPE is associated with activation of a distinct, chemokine-driven inflammatory myeloid axis, marked by expansion of CCR2^+^ classical monocytes and a coordinated cytokine response linked to endothelial dysfunction and increased vascular permeability.

## Figures and Tables

**Table 1:** Demographic and clinical characteristics of the three study groups.

Demographic or Clinical Parameters	LA-Cntrl	HA-Cntrl	HAPE	*p*-Values		
				LA-Cntrl vs. HA-Cntrl	HA-Cntrl vs. HAPE	LA-Cntrl vs. HAPE
Subjects (N)	19	47	90	-	-	-
Female/Male (N)	9/10	8/39	14/76	-	-	-
Age (years)	26.2 ± 1.46	29.3 ± 8	38.6 ± 12.45	0.1369	<0.0001	<0.001
SpO_2_ (%)	98.86 ± 0.88	91.48 ± 3.50	62.94 ± 15.15	0.5935	<0.0001	<0.0001
RR (breath/min)	18.6 ± 1.30	19.46 ± 1.87	25.84 ± 4.58	0.1064	<0.0001	<0.0001
PR (beats/min)	86 ± 7.40	85.55 ± 13.43	108.9 ± 21.30	0.8899	<0.0001	<0.0001
SBP (mmHg)	125.06 ± 14.78	119.63 ± 8.41	131.32 ± 18.42	0.0862	<0.0001	<0.0001
DBP (mmHg)	86.33 ± 15.31	79.34 ± 7.21	80.04 ± 13.25	<0.05	0.7372	0.1029
MAP (mmHg)	99.2 ± 14.54	92.77 ± 6.93	97.10 ± 12.92	<0.05	<0.05	0.5731

Data are presented as mean ± SD and are compared among the groups using an unpaired Student’s *t*-test. DBP: diastolic blood pressure; HA-Cntrl: high altitude control; HAPE: high-altitude pulmonary edema patients; LA-Cntrl: low altitude control; MAP: mean arterial pressure; PR: pulse rate; RR: respiratory rate; SBP: systolic blood pressure; SpO_2_: peripheral oxygen saturation.

## Data Availability

All data generated or analyzed during this study are included in the article and its Supplementary Information files. Other files are available from the corresponding author upon reasonable request.

## Supplementary Materials

The following supporting information can be downloaded at: https://www.mdpi.com/article/10.3390/ijms27177787/s1.

## Abbreviations

CCL7	Chemokine C-C motif ligand 7
CCR2^+^	C-C motif chemokine receptor 2^+^
cDC1	Conventional type 1 dendritic cells
cDC2	Conventional type 2 dendritic cells
cMo	Classical monocytes
CSF1	Colony stimulating Factor 1
CSF3	Colony stimulating Factor 3
CXCL11	C-X-C motif chemokine ligand 11
DBP	Diastolic blood pressure
DC	Dendritic cells
FLT3LG	FMS-related tyrosine kinase 3 ligand
HA	High altitude
HA-Cntrl	High altitude control
HAPE	High-altitude pulmonary edema
HAPH	High-altitude pulmonary hypertension
HGF	Hepatocyte growth factor
IL-10	Interleukin-10
IL-15	Interleukin-15
IL-1β	Interleukin-1 beta
IL-6	Interleukin-6
IL-7	Interleukin-7
LA	Low altitude
LA-Cntrl	Low Altitude control
MAP	Mean arterial pressure
OSM	Oncostatin M
PBMCs	Peripheral blood mononuclear cells
PCA	Principal component analysis
PH	Pulmonary hypertension
RVSP	Right ventricular systolic pressure
SBP	Systolic blood pressure
SpO_2_	Peripheral oxygen saturation
VEGFA	Vascular Endothelial Growth Factor A
