# Supplementary material for: Integrated Cytokine and Immune Cell Profiling Reveals a Distinct Immune Signature Associated with High-Altitude Pulmonary Edema
Source: Int J Mol Sci. 2026 Aug 31;27(17):7787. doi: 10.3390/ijms27177787 (PMC13565836; doi:10.3390/ijms27177787)
Supplement: Supplementary file 1 [file ijms-27-07787-s001.zip › ijms-4471160-supplementary.pdf]

Integrated cytokine and immune cell profiling reveals a distinct immune signature associated with high-altitude pulmonary edema

Authors: Singh K. *et. al*

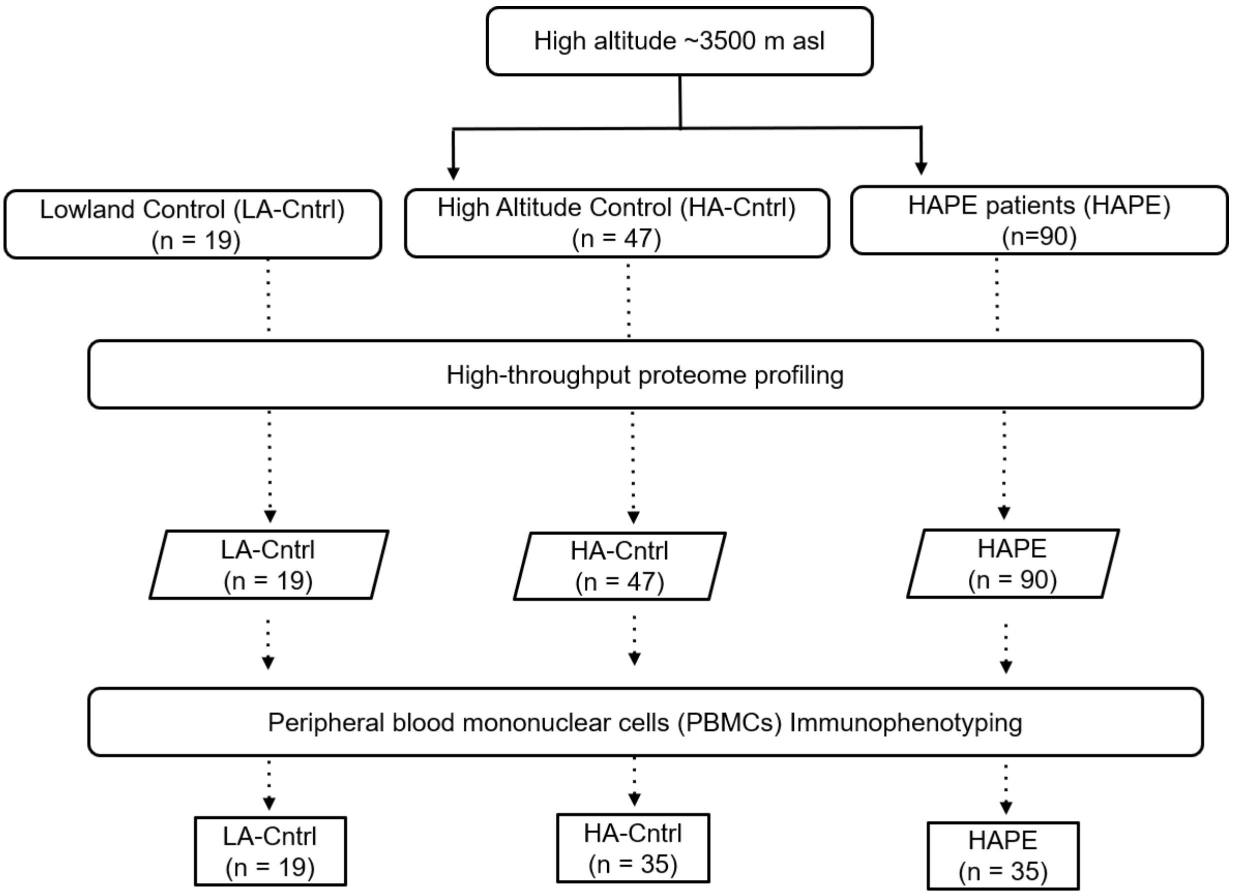

Figure S1. Schema of the study design. In the study, healthy low-altitude controls (LA-Cntrl, n=19), healthy high-altitude controls (HA-Cntrl, n=47) and patients with high-altitude pulmonary edema (HAPE, n=90) were analyzed and a subset from these samples were used for PBMCs immunophenotyping.

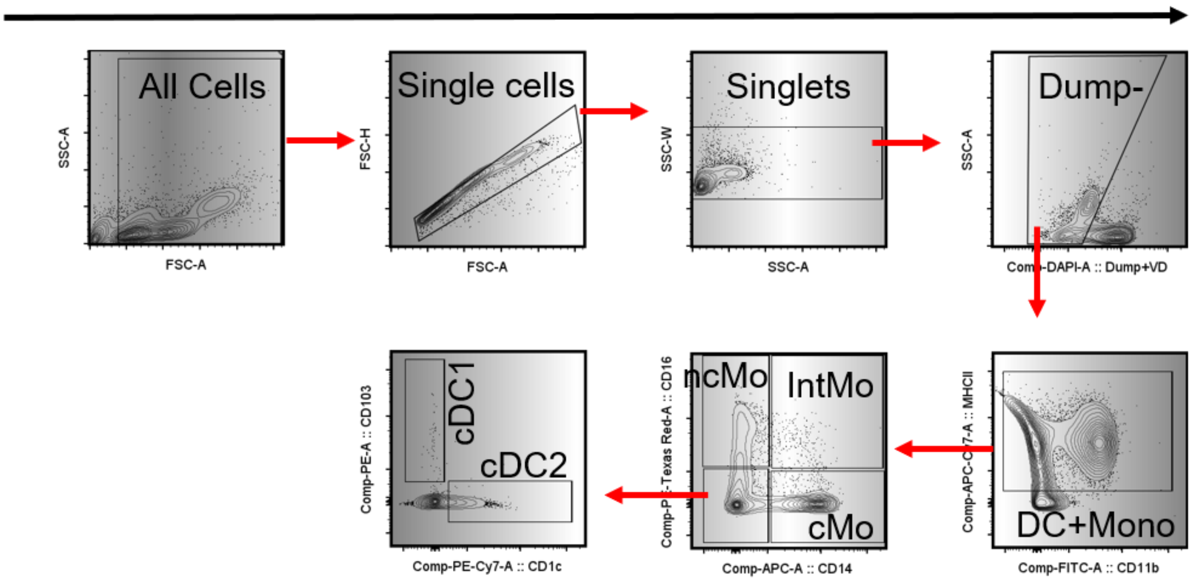

Figure S2. **Gating strategy**. The characterization of intravascular monocytes, dendritic cells and their subsets using the peripheral blood mononuclear cells.

cDC1: conventional dendritic cell type 1; cDC2: conventional dendritic cell type 2; cMo: classical monocytes; IntMo: Intermediate monocytes; DCs: dendritic cells; Mono: monocytes; ncMo: non classical monocytes

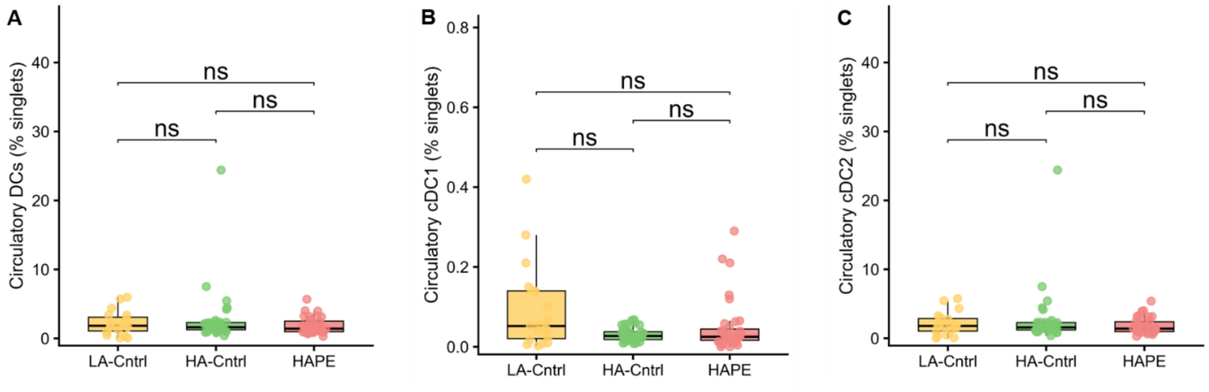

Figure S3. Quantification of intravascular dendritic cells (DCs). (A) Circulatory DC levels in the study groups. (**B**) Blunted circulatory conventional DCs subset cDC1 in the study groups. (**C**) Circulatory conventional DCs subset cDC2 in the study groups.

cDC1: conventional dendritic cell type 1; cDC2: conventional dendritic cell type 2; DCs: dendritic cells; HA-Cntrl: high altitude control; HAPE: high-altitude pulmonary edema patients; LA-Cntrl: low altitude control

Table S1. Antibodies used in the flow cytometry experiment of human biospecimen.

Antibody Specificity	Fluorochrome	Clone	Final Concentration	Manufacturer
CD3	BV421	OKT3	0.2µg	BioLegend
CD19	BV421	SJ25C1	0.2µg	BioLegend
CD335 (NK1.1)	BV421	331913	0.2µg	BioLegend
CD11b	FITC	ICRF44	0.2µg	BioLegend
CD14	APC	63D3	0.2µg	BioLegend
CD16	Dazzle 594	3G8	0.2µg	BioLegend
CCR2 (CD192)	Percp-Cy5.5	K036C2	0.2µg	BioLegend
MHCII	APC-Cy7	L243	0.2µg	BioLegend
CDc1	PE-Cy7	L161	0.2µg	BioLegend
CD103	PE	Ber-ACT8	0.2µg	BioLegend

APC: Allophycocyanin; BV: Brilliant Violet; CD: Cluster of Differentiation; Cy: Cyanine; FITC: Fluorescein Isothiocyanate; MHCII: Major Histocompatibility Complex Class II; PE: Phycoerythrin; Percp: Peridinin-Chlorophyll Protein
